# Intra-Observer and Inter-Observer Variability of Intraocular Lens Measurements Using an Interferometry Metrology Device

**DOI:** 10.3390/diagnostics14020216

**Published:** 2024-01-19

**Authors:** Benjamin Stern, Alain Saad, Roxane Flamant, Luc Joannes, Damien Gatinel

**Affiliations:** 1 Department of Ophthalmology, Rothschild Foundation Hospital, 75019 Paris, France; 2 Department of Ophthalmology, Hadassah-Hebrew University Medical Center, Jerusalem 91120, Israel; 3 Lambda-X SA, Avenue Robert Schuman, 102, B-1400 Nivelles, Belgium

**Keywords:** cataract, intraocular lens, interferometry, MTF

## Abstract

The NIMO TEMPO (Lambda-X, Nivelles, Belgium) is a novel, user-friendly and compact device designed for in vitro optical analysis of refractive and diffractive intraocular lenses (IOLs). This device analyzes the IOL wavefront and generates a synthetic eye model for numerical computation. The objective of this study was to evaluate the precision of this innovative device. Intra- and inter-observer variability were calculated using a two-way analysis of variance (ANOVA) after conducting ten measurements of eight different IOL models, with each measurement being repeated by three distinct operators (resulting in a total of 30 measurements for each IOL). The device demonstrated satisfactory intra- and inter-observer variability in evaluating IOL power and modulation transfer function (MTF) profiles, with values of 0.066 and 0.078 diopters for IOL power and 0.018 and 0.019 for MTF measurements, respectively. Furthermore, this hybrid optical and numerical in vitro IOL wavefront analyzer appears to have several advantages over conventional optical bench devices. It reduces the need for operator manipulation, and allows for numerical modeling of various optical environments, including cornea models and apertures. In conclusion, this novel metrology device designed for refractive and diffractive IOLs appears to provide a satisfactory precision, making it a promising tool in the field of IOL metrology.

## 1. Introduction

In recent years, there has been a proliferation of complex designs of intraocular lenses (IOLs), such as Extended Depth of Focus (EDOF) and multifocal lenses (MFIOL) [1], leading to a constant evolution of EDOF nomenclature with more and more subtypes based on their optical properties [2]. This complexity has resulted in a growing need for cataract surgeons and researchers to understand the optics of these lenses and compare them. However, simple paraxial optics modeling is not accurate enough to achieve this [3]. Understanding the optical properties of these IOL designs requires using advanced optical metrology devices specifically designed for IOLs, which allow for in vitro optics measurements and calculations. Compact and automated optical benches now provide ophthalmologists easier access to these experiments without requiring the complex laboratory environment and optical knowledge previously necessary to build in-house optics experiments.

In this article, we present a new device, the NIMO TEMPO (Lambda-X, Nivelles, Belgium), which is particularly interesting for ophthalmologists because it is a very compact machine that can be placed on a desk without requiring an optical bench or table. The use of the NIMO TEMPO is also made easy thanks to its user-friendly software, which allows subsequent theoretical numerical experiments based on already acquired optical wavefront data from an IOL. Powerful numerical computation also enables special features for diffractive IOLs, such as wavefront high-order aberration profile, which allows for the computation of the theoretical implant step profile from the wavefront acquisition.

This article aims to determine the precision of the NIMO TEMPO measurement by assessing intra-observer and inter-observer variability. Intra-observer variability, or repeatability, represents the error intrinsic to a single observer. On the other hand, inter-observer variability is the sum of repeatability and reproducibility, which is the error intrinsic to differences between observers [4].

## 2. Materials and Methods

### 2.1. Description of the NIMO TEMPO Device

The NIMO TEMPO (Lambda-X, Nivelles, Belgium) is an optical metrology device for refractive and diffractive IOLs. The device features an innovative wavefront sensor that allows for the rapid acquisition of the IOL’s wavefront imprint in just a few seconds. The device then employs powerful numerical computation through a dedicated software called TEMPO-MENTOR and an external computer, which enables the application of different corneal eye models and aperture sizes.

**Figure 1:**
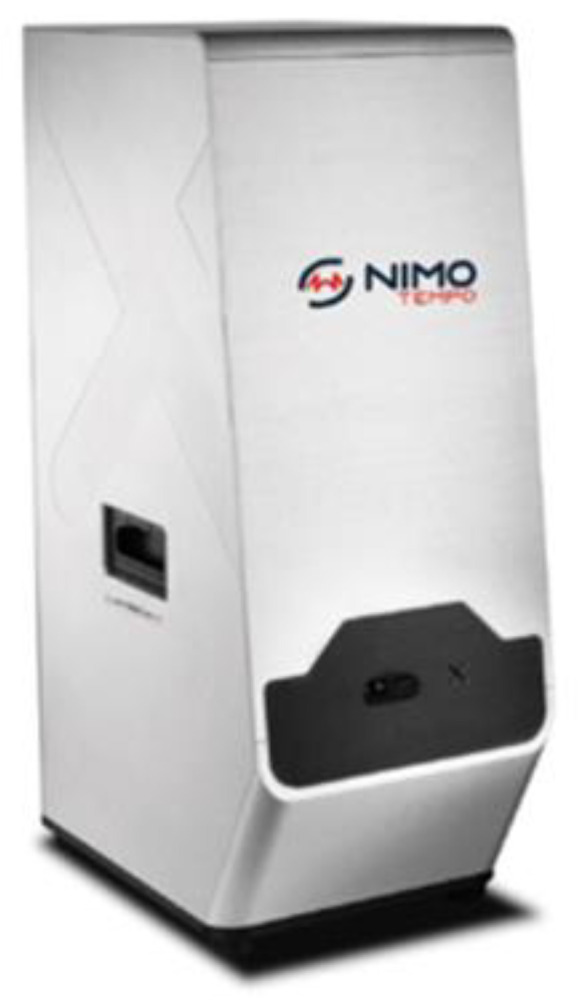
Photograph of the NIMO TEMPO Instrument.

The NIMO TEMPO (Lambda-X, Nivelles, Belgium) can be used by IOL manufacturing companies for production control or by individuals, such as ophthalmologists researching to gain insights into the optical quality of the intraocular lenses they implant. The device is designed to be compact, and it can be placed on a normal desk or in a doctor’s office without requiring a special optical table (see Figure 1).

**Figure 2:**
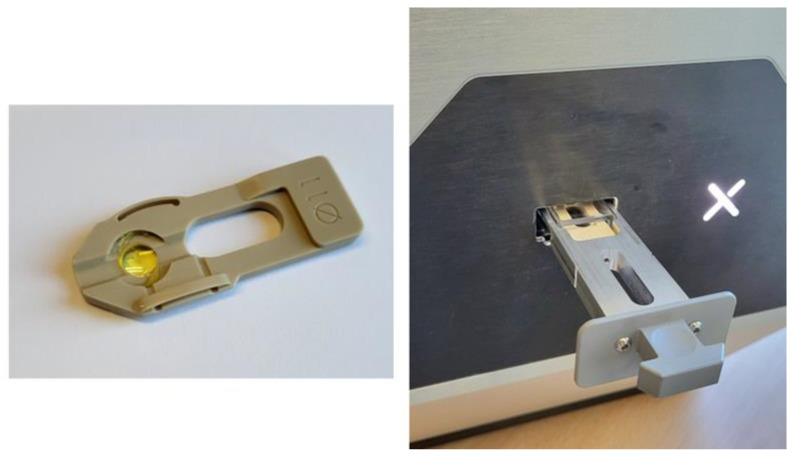
IOL in its holder (**left**) and wet cell in the injector (**right**).

The intraocular lens under test is placed upside down into water or saline solution in a quartz cell. It is introduced into an injector (see Figure 2) via a holder that prevents the IOL from moving and ensures it is well placed without tilting.

Measuring 3-piece IOLs can be challenging due to the small size of the haptics, which can cause the lens to tilt. Moreover, haptic angulation can theoretically cause the lens to be more anterior, affecting power calculation. Hydrophilic lenses can also be difficult to measure due to their high adherence to water when placing and removing the holder in water, which can cause the IOL to dislocate from the measurement location.

Knowledge of the IOL material and solution refractive indices is crucial for conducting accurate measurements. Fortunately, obtaining this information is not problematic, as these parameters are typically provided by the manufacturer or can be measured using a refractometer. On the other hand, the user is responsible for defining the lens geometry. If the defined geometry does not perfectly match the actual lens geometry, any discrepancies will be addressed via the residual high order aberrations (see below for details). It is important to note that the assumption of the lens having a biconvex profile is made. Consequently, measuring newly developed IOLs with a distinctive inverted meniscus shape, such as ArtIOLs (Voptica) [5], can present challenges.

### 2.2. Measurement Principle

The optical performance of IOLs is typically evaluated using optical benches. These benches consist of a physical cornea model placed before the IOL in the optical path to allow for direct measurement of image quality in the image plane using a microscope. The Modulation Transfer Function (MTF) is then calculated for different frequencies of spatial resolutions using the Point Spread Function (PSF). For a reminder, the MTF describes the contrast degradation of a sinusoidal pattern as it passes through the lens [6].

**Figure 3:**
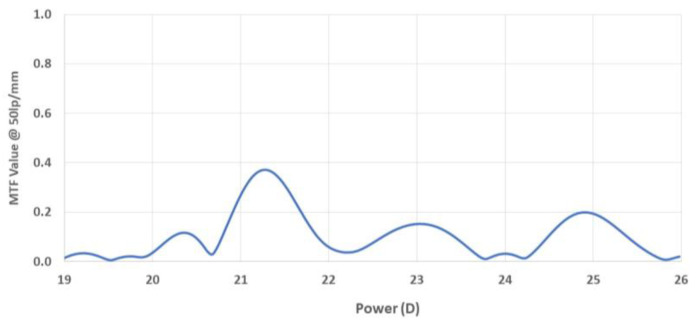
Through-focus MTF curve of a trifocal intraocular lens obtained from NIMO TEMPO data. Three peaks can be observed, each representing a focus.

For a constant spatial frequency, the Through-Focus MTF curve can be calculated to represent the value of the MTF across a range of planes around the focal plane (see Figure 3).

The NIMO TEMPO (Lambda-X, Nivelles, Belgium) is a unique device that differs from the classic optical bench due to its interferometer, which captures the Optical Path Difference (OPD) map after the light has passed through the IOL. The image quality is then calculated by synthetic wavefront propagation up to the focus plane(s), and the modulation transfer function (MTF) and through-focus MTF (TF-MTF) in each plane around the focus are digitally computed from a single wavefront acquisition.

**Figure 4:**
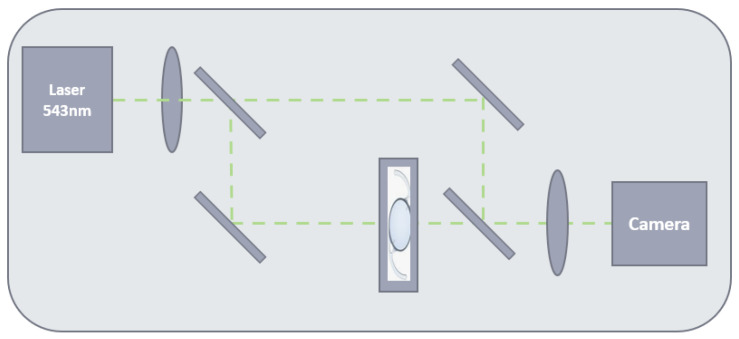
NIMO TEMPO Components diagram.

The NIMO TEMPO is composed of three parts: a 543 nm laser source and its illumination optics, a Mach-Zehnder interferometer, along with imaging optics equipped with a camera (See Figure 4).

An interferometer is an optical device that splits one laser light beam into two rays and captures the interference pattern of those beams when brought back together. By placing the lens to be measured on one of these beams’ trajectories, one can evaluate its optical characteristics based on the deformation of the interference pattern it creates. The NIMO TEMPO detects the optical center of the lens and provides automatically centered measurements, which facilitates ease of use. However, it may have limited flexibility for experiments involving decentration effects.

### 2.3. NIMO TEMPO Results

#### 2.3.1. Through-Focus MTF Results

As previously discussed, the NIMO TEMPO (Lambda-X, Nivelles, Belgium) numerically propagates the wavefront to the image plane along the focal axis. It calculates the MTF at each location to generate a TF-MTF curve. The TF-MTF is calculated for two meridians (X and Y), representing the flattest and steepest meridians, respectively.

The MTF depends on spatial frequency, and the TF-MTF is generated for a specific spatial frequency, with 50 lp/mm being the most commonly used frequency in the literature. The MTF is also dependent on aperture sizes, and the aperture size should be settled for calculating the TF-MTF curve, with 3 mm and 4.5 mm being the most commonly used to simulate photopic and scotopic conditions. However, it is important to note that the aperture size is not equivalent to the entrance pupil size, which is magnified by 13.3% by the cornea for a standard eye [3]. Therefore, an aperture size of 3 mm and 4.5 mm will correspond to an entrance pupil size of 3.4 mm and 5.1 mm, respectively. Additionally, caution should also be exercised to differentiate between preoperative and postoperative pupil sizes, as pupil size is known to generally reduce after cataract surgery [7].

Several parameters can be deduced from the TF-MTF curve. The Spherical Equivalent Power (SEP) can be determined by averaging the powers of the main MTF peaks of the two meridians, X and Y. The difference in power between these two peaks indicates the cylinder power of the lens. It is important to note that the lens’s tilt may also cause a difference between the MTF of the two meridians, which could be falsely interpreted as cylinder power. Add powers are identified as additional MTF peaks at higher dioptric powers in the Through-Focus MTF curves at 50 lp/mm, with the MTF of these peaks representing the Add Focus MTFs. For toric IOLs, the cylinder axis is determined from the computed modulation map and compared to the label marks to determine the label axis error in degrees.

In this study, we analyzed the through-focus curves using a 3 mm aperture for two commonly employed spatial frequencies: 50 and 25 lp/mm. Notably, according to ISO 11979-2:2014 guidelines [8], multifocal IOLs should be assessed at 50 lp/mm. Moreover, we selected 25 lp/mm as it represents the midpoint within the range of 1 to 50 lp/mm. This range is commonly used to evaluate the modulation transfer function area (MTFa) that has garnered increasing interest and may predict the visual acuity of the patients after implantation [9,10].

The wavefront acquisition data can be interpreted with or without a synthetic numerical eye model, including different models established according to ISO 11979-2 recommendations [8]. In this study, we conducted all the analyzes without a synthetic eye model to assess the raw data recorded by the machine.

#### 2.3.2. Residual High Order Aberrations and Diffractive Steps Reconstruction

**Figure 5:**
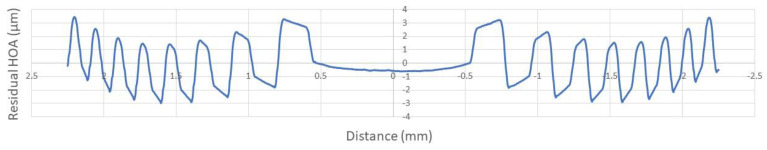
IOL diffractive step profile extracted from the measured wavefront.

The unique design of the NIMO TEMPO (Lambda-X, Nivelles, Belgium) allows for the capture of the wavefront that exits the IOL, enabling access to important characteristics of the IOL, such as asphericity, toricity, and discrete refractive or diffractive elements. Through an iteration process, the wavefront acquisition is compared to a ray-tracing lens model that can generate a wavefront matching the measured wavefront. The residual high-order aberrations, that may result from manufacturing errors or diffractive steps, are also computed in this process. In the case of a diffractive profile, the residual high-order aberration can be computed to show a hypothetical diffractive steps profile that matches the residual high-order aberration wavefront. This unique process permits access to valuable information about diffractive designs of different IOLs (see Figure 5). The manufacturer validated this process with Zemax^®^ software. In this study, we did not assess the variability of this modality as being a beta version and requiring more understanding about its significance.

### 2.4. Intra-Observer and Inter-Observer Variability Method of Assessment

Ten in vitro wavefront measurements were acquired for eight different IOLs by three distinct operators (for a total of 30 measurements for each IOL). Details of IOLs can be seen in Table 1. The measurements were conducted according to the same protocol for each IOL at Rothschild Foundation Hospital by three ophthalmologists: the lead author and two co-authors of this manuscript. The lead author administered a 30 min training session on the operation of the machinery to the co-authors before the measurements were taken. A two-way ANOVA was used to determine the intra-observer and inter-observer variability as the intra-observer SEM (Standard Error of Measurement), also called repeatability and inter-observer SEM, respectively, for the spherical equivalent power (SEP) of far focus, cylinder power, label axis error, add power, and MTF at 25 lp/mm and 50 lp/mm at different MTF peaks. The statistical analysis of added foci, including power and MTF, was conducted collectively for both intermediate and near foci for the trifocal lenses.

The intra-observer SEM was defined as follows:

$${SEM}_{intra}=\sigma_{equipment}=\sqrt{{Mean\;Square}_{equipment}}$$

When $\sigma_{equipment}$ represents the standard deviation associated with the equipment and ${Mean\;Square}_{equipment}$ is the mean deviation of all trials for a given part and given technician from the average for that part and technician [11].

The inter-observer SEM is defined as:

$${SEM}_{inter}=\sqrt{\sigma_{equipment}^{2}+\sigma_{observer}^{2}+\sigma_{interaction}^{2}}$$

If the interaction was not statistically significant according to the ANOVA test with α < 0.05, then the variance related to the interaction, $\sigma_{interaction}^{2}$, was omitted.

We also defined the Gage R&R, a parameter used in the industry to characterize the precision of measurement devices. It is expressed as a percentage and is defined as:

$$Gage\;R\&R=\frac{\sigma_{error}^{2}+\sigma_{observer}^{2}+\sigma_{interaction}^{2}}{\sigma_{Total}^{2}}*100$$

A Gage R&R of less than 1% is considered acceptable for a measurement system, while a Gage R&R of more than 9% is considered unacceptable. In this study, we compared our results to the manufacturer’s Gage R&R study, in which Lambda-X measured 18 IOLs (3 monofocal, 5 toric, 5 multifocal, and 5 multifocal toric) using 3 observers.

We also calculated the Minimum Detectable Difference (MDD) for each parameter, which is the minimum statistically significant detectable difference that can be considered unrelated to measurement error (with a 95% confidence interval) between two lenses, as defined by Popovic and Thomas [4]:

$$MDD=1.96\times \sqrt{2}SEM=2.8\;SEM$$

## 3. Results

The intra-observer SEM (repeatability) and inter-observer SEM for the spherical equivalent power (SEP) of the intraocular lens (IOL), cylinder power, and add power were 0.066 D and 0.078 D, 0.046 D and 0.046 D, and 0.012 D and 0.012 D, respectively. The gage R&R of these parameters was very low, well below the 1% acceptable threshold. These parameters’ minimum detectable difference (MDD) were 0.216 D, 0.127 D, and 0.034 D, respectively.

Regarding the modulation transfer function (MTF), the intra-observer SEM and inter-observer SEM for 50 lp/mm at far and add focus were 0.018 and 0.019, and 0.0034 and 0.004, respectively, and for 25 lp/mm, 0.026 and 0.026, and 0.007 and 0.007, respectively. The Gage R&R of the MTF results is higher than that of the power results, and is slightly above the 1% threshold, but well below the 9% unacceptable error of measurement. These results are summarized in Table 2. In the supplementary data (Table S1), a table is provided that presents the mean values from 30 measurements, which were conducted by all operators on eight IOLs, and includes the corresponding standard deviations.

## 4. Discussion

In recent years, cataract surgery has evolved from treating a potentially blinding disease to a refractive method of improving living conditions [12], especially in patients with early cataracts and presbyopia. Ophthalmologists strive to provide the best refractive outcomes to their patients by using advanced biometry and IOL formulas, attempting to comprehend the impact of each stage of cataract development on refractive results, and utilizing the latest intraocular lens technology, including EDOF and multifocal IOLs. However, most ophthalmologists lack the necessary optics background to grasp these new technologies fully and often rely on IOL manufacturers’ narratives and experiments, which are typically commercial and lack transparency regarding IOL geometry [13]. Furthermore, ophthalmologists frequently rely significantly on IOL labeling, inadvertently disregarding the potential consequences of manufacturing irregularities on refractive errors.

In this article, we described the NIMO TEMPO, a desk-usable IOL metrology machine that is easy to use, fast, and, most importantly, provides satisfactory repeatability and precision for assessing IOL power and MTF profiles. The inter-observer SEM for IOL power was 0.078 D, and the minimum detectable difference in IOL power was 0.216 D. Additionally, we demonstrated that the device could provide precise MTF measurements in the far and add foci.

This device utilizes wavefront sensor technology and offers certain advantages over classic IOL optical bench machines. Its hybrid optical and powerful numerical process combines ray-tracing model technology and optical wavefront acquisition, allowing for flexible possibilities of IOL experiments. In the future, this technology would probably enable the development of a better understanding of IOL optics in complex environments. While not assessed in this study, the NIMO TEMPO also gives novel information that was previously unattainable with conventional optical benches. It allows for the acquisition of the residual high-order aberration profile, thereby providing valuable insights into IOL geometry.

The International Standardization Organization’s (ISO) norm from 1999 (ISO-11979), revised in 2014 [8], provides guidelines concerning optical properties and testing methods. It specifies power tolerance for IOL labeling by manufacturers, which is related to the power of the lens and is particularly forgiving for high-power lenses. Specifically, the allowed tolerance is ±0.3 D for IOL power from 0 to 15 D, ±0.4 D from 15 to 25 D, ±0.5 D from 25 to 30 D, and 1.0 D over 30 D. These standards, rooted in Norrby’s 1996 study [14], have remained unchanged since 1999. However, in the contemporary context, a ±1 dioptric power error in a lens, for example, of +32.0 D is generally considered unacceptable. Indeed, the precision of refractive outcomes in cataract surgery has seen rapid improvements since 1996, facilitated by advancements in optical biometry, enhanced power calculation formulas, and surgical techniques such as small corneal incisions. Studies conducted between 1996 and 2005 found that 44.6–58.4% of patients achieved a deviation from the target refraction of ±0.5 D, a proportion that increased to 61.2–88.0% between 2007 and 2017 [15]. However, even with the best efforts, reaching 100% of eyes within ±0.5 D remains an utopic goal, given that post-operative refraction measurement error has a 95% test–retest spherical equivalent measurement of approximately ±0.5 D [15].

Despite these advancements, some of these errors may still arise due to inaccuracies in IOL labeling. While manufacturers may already adhere to stricter standards than the ISO norm, it appears that further refinement of the ISO norm itself may be necessary. Our study suggests that hybrid technologies, such as NIMO TEMPO, can provide precise measurements. Although additional research is necessary to validate its accuracy, if confirmed, the permissive guidelines could be subject to revision. In fact, measurement errors in interferometry may not be correlated with the power of the lens, unlike traditional optical benches. Therefore, it could be feasible to establish a uniform standard for all IOL powers.

Furthermore, due to improved precision of refractive outcomes and quality control, as well as the capability to differentiate between two lenses with a difference of only 0.216 D (minimum detectable difference, MDD) using the NIMO TEMPO, manufacturers are theoretically capable of offering quarter-diopter power increments in their range of lenses, and some manufacturers have already implemented this, such as Lenstec and Cutting Edge.

In the field of metrology devices for IOLs, limited literature is available. In vitro studies have been conducted using two main standard optical bench devices for IOLs mentioned in the literature: the OptiSpheric IOL PRO2 (Trioptics GmbH, Wedel, Germany) and the PMTF (Lambda-X, Nivelles, Belgium). In addition to these devices, other wavefront sensors for in vitro analysis of IOLs are also available. For example, the WaveMaster (Trioptics) uses Hartman Shark technology [16]. However, this device, unlike the NIMO TEMPO, is unable to measure diffractive IOLs.

The NIMO TEMPO technology has certain limitations. Firstly, the NIMO TEMPO uses a specific laser frequency (543 nm) and not white light, which prevents the measurement of polychromatic aberrations. This limitation is not exclusive to wavefront acquisition technology, as most optical benches use laser technology to avoid internal chromatic aberrations of the device. Only a few studies have been conducted on experimental devices permitting the determination of white light MTF [17,18]. Secondly, the NIMO TEMPO currently does not allow for off-axis and tilted IOL measurements. This limitation could be significant for research purposes, given that the eye is not a perfectly centered optical system. In a patient’s eye, the IOL, after implantation, is likely to be positioned with a slight tilt and decentration. Moreover, it is well documented that such tilt and decentration can have varying impacts on optical quality, depending on IOL geometry [19,20]. However, the NIMO TEMPO may have the capability to simulate tilt and decentration in the future through a simple software update, owing to its numerical component and synthetic eye model. Finally, a common limitation shared by many metrology devices is that the IOL placed in water is held in position by its haptics, which can lead to potential movement or improper placement, resulting in tilt or anterior displacement and subsequently causing measurement errors.

Our study also has some limitations. First, it is important to note that we only evaluated the device’s precision and did not assess its accuracy compared to the gold standard measurement. The manufacturer guarantees an accuracy of 0.5% of the power and 0.03 MTF units, as verified through the measurement of certified glass lenses. Second, it is necessary to cautiously approach our assumptions regarding the ISO norm, as these norms need to be accurate for the number of laboratories using different machines. Third, we used eight different lenses to determine the R&R, slightly lower than the 10 lenses typically recommended for the ANOVA Gage R&R study. However, we compensated for this by taking many measurements (10) per observer and IOL. Fourth, we checked the precision of the machine for IOLs between 8 and 30 D, recognizing that the wavefront acquisition could be less accurate for low-power IOLs. Fifth, due to the low number of toric lenses available, the precision of the cylinder direction could not be evaluated in comparison to the labeled axis, which is exclusively provided for toric lenses. Finally, we analyzed MTF X and Y together and the Add power of the intermediate and near focus together.

Future research needs to be conducted to determine the level of agreement between wavefront acquisition metrology technology for IOLs and conventional optical benches. In addition, the repeatability and precision of IOL measurements should be studied for low- and negative-power lenses.

In conclusion, the advancements in metrology devices and the availability of precise desk-mounted devices that can be operated by ophthalmologists, such as the NIMO TEMPO, hold the potential to enhance our understanding of complex IOLs. This opens the door for convenient in vitro research that can be conducted with relative ease, serving as a theoretical basis for clinical research. Furthermore, it appears that the high precision offered by emerging metrology technologies in the industry will impact future IOL manufacturing standards, allowing for higher quality control standards.

## Figures and Tables

**Table 1:** Intraocular lenses parameters.

Piece #	IOL Brand Name	IOL Type	Power (D)	IOL Refractive Index	Approx. Thickness (mm)
1	Johnson and Johnson Tecnis DCB00	Monofocal	+20.0	1.47	0.6
2	Johnson and Johnson Tecnis DCB00	Monofocal	+34.0	1.47	0.9
3	Alcon Acrysof IQ Vivity	EDOF	+21.5	1.55	0.7
4	Alcon Acrysof Restor Toric	Diffractive Bifocal Toric	+28.0/Cyl 1.0 Add +3.0	1.55	0.7
5	Alcon Acrysof SN60T3	Monofocal toric	+22.0/Cyl 1.50	1.55	0.7
6	Johnson and Johnson Tecnis Eyhance	Enhanced monofocal	+8.0	1.47	0.5
7	BVI PhysIOL Finevision MicroF	Diffractive Trifocal	+19.0	1.46	0.85
8	BVI PhysIOL Finevision MicroF	Diffractive Trifocal	+19.0 Add +3.5	1.46	1

IOL: Intraocular lens, D: Diopter, Approx.: Approximate, mm: millimeter, Cyl: cylinder, EDOF: Extended Depth of Focus.

**Table 2:** Intra- and inter-observer variability of IOL parameters.

Parameters	Intra-Observer SEM (CI 95%)	Inter-Observer SEM	Gage R&R	Manufacturer Inter-Observer SEM	Minimum Detectable Difference (MDD)
SE Power (D)	0.066 (0.047–0.085)	0.078	0.01%	<0.04	0.216
Cylinder (D)	0.046 (0.042–0.050)	0.046	0.6%	<0.02	0.127
Add Power (D)	0.012 (0.010–0.013)	0.012	0.02%	<0.03	0.034
Far Focus MTF50	0.018 (0.015–0.022)	0.019	1.5%	0.015	0.053
Add Focus MTF50	0.0034 (0.0026–0.0043)	0.004	1.5%	0.015	0.012
Far Focus MTF25	0.026 (0.024–0.028)	0.026	1.2%	0.015	0.072
Add Focus MTF25	0.007 (0.0064–0.0076)	0.007	2.6%	0.015	0.019

SEM: Standard Error of Measurement, CI: Confidence Interval, R&R: Repeatability & Reproducibility, MDD: Minimal Detectable Difference, SE: Spherical equivalent, D: Diopter, MTF: Modulation Transfer Function.

## Data Availability

The original contributions presented in the study are included in the article/supplementary material, further inquiries can be directed to the corresponding author/s.

## Supplementary Materials

The following supporting information can be downloaded at: https://www.mdpi.com/article/10.3390/diagnostics14020216/s1, Table S1: Mean and standard deviations of IOL measurements.
