# Supplementary material for: Intra-Observer and Inter-Observer Variability of Intraocular Lens Measurements Using an Interferometry Metrology Device
Source: Diagnostics (Basel). 2024 Jan 19;14(2):216. doi: 10.3390/diagnostics14020216 (PMC10813881; doi:10.3390/diagnostics14020216)
Supplement: Supplementary file 1 [file diagnostics-14-00216-s001.zip › diagnostics-2717751-supplementary.pdf]

<u>Table S1 :</u> Mean and standard deviations of IOL measurements.

Parameters		IOL n°1	IOL n°2	IOL n°3	IOL n°4	IOL n°5	IOL n°6	IOL n°7	IOL n°8
SE Power (D)		20.179±0.047	35.622 ± 0.083	21.829 ± 0.028	28.281 ± 0.052	22.612 ± 0.064	7.846 ± 0.130	18.964 ± 0.040	22.723 ± 0.093
Cylinder (D)		0.069 ± 0.013	0.042 ± 0.027	0.063 ± 0.024	1.006 ± 0.029	1.583 ± 0.0306	0.168 ± 0.107	0.082 ± 0.025	0.134 ± 0.034
Add Power (D)		-	-	-	3.057 ± 0.006	-	-	I: 1.608 ± 0.005<br>N: 3.356 ± 0.011	I: 1.596 ± 0.005<br>N: 3.402 ± 0.024
Far Focus MTF50	X-axis	0.400 ± 0.006	0.608 ± 0.051	0.238 ± 0.008	0.294 ± 0.008	0.481 ± 0.017	-	0.171 ± 0.006	0.197±0.009
	Y-axis	0.404 ± 0.008	0.608 ± 0.035	0.237 ± 0.006	0.292 ± 0.010	0.482 ± 0.015	-	0.170 ± 0.008	0.193±0.006
Add Focus MTF50	X-axis	-	-	-	0.155 ± 0.004	-	-	I: 0.058 ± 0.004<br>N: 0.101 ± 0.003	I: 0.096 ± 0.004<br>N: 0.129 ± 0.005
	Y-axis	-	-	-	0.156 ± 0.005	-	-	I: 0.059 ± 0.005<br>N: 0.099 ± 0.002	I: 0.096 ± 0.004<br>N: 0.130 ± 0.004
Far Focus MTF25	X-axis	0.656 ± 0.01	0.782 ± 0.049	0.280 ± 0.007	0.382 ± 0.007	0.718 ± 0.024	0.175 ± 0.054	0.227 ± 0.011	0.285 ± 0.009
	Y-axis	0.653 ± 0.013	0.788 ± 0.025	0.281 ± 0.009	0.382 ± 0.008	0.716 ± 0.020	0.156 ± 0.049	0.225 ± 0.010	0.287 ± 0.022
Add Focus MTF25	X-axis	-	-	-	0.213 ± 0.006	-	-	I: 0.114 ± 0.007<br>N: 0.200 ± 0.004	I: 0.124 ± 0.006<br>N: 0.187 ± 0.007
	Y-axis	-	-	-	0.211 ± 0.005	-	-	I: 0.114 ± 0.012<br>N: 0.198 ± 0.005	I: 0.121 ± 0.007<br>N: 0.185 ± 0.007

D : diopter, I: Intermediate, MTF: Modulation Transfer Function, N: Near, SE : Spherical equivalent.
